# Effectiveness of an intervention program to improve the detection of cognitive impairment in LMIC setting

**DOI:** 10.1002/alz.086012

**Published:** 2025-01-09

**Authors:** Juan J. Llibre‐Rodriguez, Arianne Rodriguez‐Velasco, Raul Gutierrez‐Herrera, Arianna Almirall‐Sanchez, Tania Zayas‐Llerena, Milagros Guerra‐Hernandez, Jorge J. Llibre‐Guerra, DAVOS Alzheimer Collaborative

**Affiliations:** ^1^ Dementia Research Unit/Medical University of Havana, Havana, Havana Cuba; ^2^ School of Public Health, Havana Cuba; ^3^ Universidad de Nuevo León, Monterrey, EM Mexico; ^4^ Global brain health institute ‐ Trinity College, Dublin Ireland; ^5^ Trinity College, Dublin Ireland; ^6^ Dementia Research Unit/Medical University of Havana, Havana Cuba; ^7^ DIAN‐TU.Knight Alzheimer’s Disease Research Center, St. Louis, MO USA; ^8^ Global Brain Health Institute, University of San Francisco California, San Francisco, CA USA; ^9^ Washington University in St. Louis, School of Medicine, St. Louis, MO USA

## Abstract

**Background:**

While two‐thirds of the 57 million people with dementia worldwide live in low‐ and middle‐income countries, research to inform dementia‐related policy in these regions remains scarce. We aimed to increase early dementia diagnosis and the use of standardized cognitive assessment by PCP in primary care settings in Cuba

**Method:**

We selected 16 Primary care clinics, with an estimated 160‐200 providers. Clinics were carefully paired based on a number of providers, population demographics, and baseline rates of cognitive impairment diagnosis before being randomized into two groups: one to receive a targeted intervention and the other to continue with usual care practices. The target intervention included a dementia training program and methods for cognitive assessment. A questionnaire about attitudes and practices towards the use of technology‐screening tools, diagnosis, and management of cognitive impairment in primary care was applied to GP at the beginning of the study, 6 months and 12 after training course. The primary endpoint was change in dementia diagnosis rates 12 months after the target intervention.

**Result:**

Compared with the usual care group, the intervention group showed significant statistical improvement in the level of confidence in their ability to engage in key aspects of the neurocognitive evaluation, perform memory screening in older people during annual health visits, and confidence during cognitive evaluation. In addition, the intervention group showed significant improvement in interpreting the diagnostic test (χ² = 8.022; p‐value = 0.004), selecting which test is appropriate (χ² = 10.756; p‐value = 0.001) and familiarity with diagnostic criteria for different forms of dementia. In the intervention group, the frequency of PCP cognitive diagnoses (primary outcome) and referrals to memory specialists increase by 141% relative to baseline, and the use of standardized cognitive assessment for annual cognitive evaluation increased by 83%. The overall prevalence of dementia at selected primary care clinics was 10.6% (95% CI, 9.3%‐12.8%).

**Conclusion:**

This study confirms the feasibility of training primary care physicians (PCPs) for the early detection, treatment, and support of individuals with dementia and their families.

